# Supplementary material for: Graph deep learning locates magnesium ions in RNA
Source: QRB Discov. 2022 Oct 6;3:e20. doi: 10.1017/qrd.2022.17 (PMC10249658; doi:10.1017/qrd.2022.17)
Supplement: Supplementary file 1 [file qrdsup.zip › S2633289222000175sup001.pdf]

Supplementary materials: Graph deep learning locates magnesium ions in RNA

Yuanzhe Zhou[1] and Shi-Jie Chen[2,*]

[1] Department of Physics and Astronomy, University of Missouri at Columbia, Columbia, MO 65211-7010, USA
[2] Department of Physics and Astronomy, Department of Biochemistry, Institute of Data Sciences and Informatics, University of Missouri at Columbia, Columbia, MO 65211-7010, USA
[*]To whom correspondence should be addressed. E-mail: chenshi@missouri.edu

Supplementary Information

Curating the set of high-quality Mg^{2+} sites

The high-quality Mg^{2+} binding sites are clustered from the MgRNA benchmark set (Zheng et al., 2015). The original MgRNA benchmark set contains 15334 high-quality sites extracted from 489 PDB files, among which, 14682 sites and 652 sites are collected from 294 ribosomal structures and 195 non-ribosomal structures, respectively. In order to remove the redundancy in the MgRNA benchmark set, we cluster all the sites according to the structural similarity between their neighboring RNA segments (defined as the nucleotides within 10 Å of the site). We use 3 Å as the RMSD cutoff for the clustering, and only one representative ion site is selected for each cluster. This leads to a list of 1974 non-redundant high-quality ion binding sites. For each high-quality ion binding site, only top-7 predictions of the MgNet model are used to evaluate the performance (roughly the number of Mg^{2+} required to neutralize the negative charges of the nearby RNA segments). Details of the high-quality set can be found in Supplementary Dataset S1. We choose the MgRNA dataset because the RNA-bound Mg^{2+} sites in MgRNA benchmark set are comprehensively validated and filtered, and are considered to be 'reliable' experimental data. We randomly group the (high-quality) dataset into five subsets, with the same total number of experimentally determined Mg^{2+} sites in each subset except for one subset with 394 total sites. For each ion binding site in the high-quality set, the space covered by the associated 3D images around the corresponding nucleotides are used by the MgNet model to predict ion distributions (Supplementary Fig. S2).

Collecting data for motif identification

RNA structures used in motif identification are collected from nucleic-acid database (NDB). Initially, 980 crystallographically determined Mg^{2+}-containing structures with resolution better than 3 Å are downloaded. To avoid the redundancy in the dataset, we reduce the 980 Mg^{2+}-containing structures to 350 crystal structures with 373 representative sequence/structure equivalence classes according to the representative set of RNA 3D structures (Leontis and Zirbel, 2012). Details of the high-quality set can be found in Supplementary Dataset S1.

Defining 3D image

We use 24 Å $\times$ 24 Å $\times$ 24 Å cubic boxes to capture the information from binding and non-binding sites. The information contained in these boxes serves as the input "images" for deep learning. Similar to a 2D image having three color channels (red, green, blue), our 3D images contain two feature channels, volume occupancy and partial charge (Supplementary Table S10). For each channel in an image, there are $48 \times 48 \times 48$ voxels (pixels for 3D images), and each voxel has a volume of 0.5 Å $\times$ 0.5 Å $\times$ 0.5 Å. As a result, a 3D image is generated by two $48 \times 48 \times 48$ sized boxes stacked together.

Generating 3D images for RNA

For a given structure, each nucleotide is associated with an image. The midpoint between the backbone carbon atom C1$'$ and the base nitrogen atom connected to the C1$'$ atom is used as the origin for the corresponding image box. A local Cartesian coordinate system associated with each residue is set to avoid the need of image augmentation (i.e., 3D rotation transformation for each image). The space around the residue (within the image box) is discretized and filled with voxel values. The local coordinate system is set up according to the following steps. First, we select three

key atoms in a residue: O4′ and C1′ from the sugar ring and one nitrogen atom from the base (N1 from uracil and cytosine or N9 from adenine and guanine, see Supplementary Fig. S1a). Second, we calculate the vectors from C1′ to O4′ (**CO**) and from C1′ to the base nitrogen atom (**CN**). We select vectors **CN** and $\mathbf{CN} \times \mathbf{CO}$ as the x- and the z-axis, respectively. The cross product of the z- and x-axes gives the y-axis.

We fill images with voxel values according to the Van der Waals radius r_{vdw} of each atom type. For each voxel in a property channel, we go through all RNA atoms to calculate the voxel occupancy. For example, we first calculate the distance r_{ij} between the RNA atom j and a given a voxel i. Then, we use a step-like function

$$n_i = f_j \times (1 - e^{-(\frac{r_{vdw}}{r_{ij}})^{12}}) \tag{1}$$

to evaluate the contribution of RNA atom j to the voxel value, where f_j represents the feature value associated with atom j. For the volume occupancy channel, f_j is 1, whereas for the partial charge channel, f_j is the partial charge of atom j. If more than one RNA atom contributes to the same voxel, we assign the average value from the contributors (Doerr et al., 2016).

In total, we generate 15912 images for the 177 structures. In the training process, we remove images with less than 300 non-zero voxels from the training set.

Labeling targets

Because training MgNet is a supervised learning task, we need to label each image with its true ion distribution and use image-label pairs to guide the learning process. In reality, the precision of ion positions in RNA structures is limited due to various factors. For example, X-ray diffraction can only resolve ion positions up to a certain resolution. In order to take these factors into consideration, we employ the distribution function in Eq. 1 (with $r_{vdw} = 2.5$Å for Mg^{2+}) to account for the diffusiveness of the experimentally observed Mg^{2+} ions. The distribution of Mg^{2+} within each image box is used as the target label in MgNet training to compute the mean squared error (MSE) loss per voxel between the true and the predicted distributions. The minimization of the MSE loss guides the parameter training process in MgNet.

Choosing hyperparameters for MgNet

MgNet uses the two-channel 3D images of the RNA as the input and outputs a predicted Mg^{2+} distribution for each image. The network has 22 convolutional layers. Each of the first 21 layers contains 16 $3 \times 3 \times 3$ filters, and the last layer has only one $3 \times 3 \times 3$ filter. We use 16 filters in each layer to optimize the usage of the GPU memory and the computer time spent on the training. Following a previous study (Ioffe and Szegedy, 2015), we apply the batch normalization in each layer immediately after the convolutional operation and before the ‘Rectified Linear Unit’ (Nair and Hinton, 2010) activation. We also apply the batch normalization (Ioffe and Szegedy, 2015) for the last layer before the final activation, and replace the ‘Rectified Linear Unit’ (Nair and Hinton, 2010) activation function with a sigmoidal activation function to keep the predicted voxel value in the range from 0 to 1. Based on the plain network, we insert residual shortcut connections for every block with two hidden layers. The shortcut takes an identical input from a previous block and maps the identity shortcut right before the activation of the second hidden layer within the block (Supplementary Fig. S3 and Fig. S4). We initialize the weights (He et al., 2015, 2016) and train all residual nets from scratch. To keep the input and output image sizes identical, we do not use any downsampling methods during the training.

The only data preprocessing used is the subtraction of the voxel mean from each image. For a given channel, the voxel mean is calculated by averaging the training set voxel values for all possible voxel positions in the corresponding channel. To center the data, we subtract the voxel mean from each voxel value. we perform this preprocessing for the training, validation, and test sets.

For the network optimizer (Kingma and Ba, 2017), we use default parameters provided by PyTorch (Paszke et al., 2019) for momentum scheduling ($\beta_1 = 0.99, \beta_2 = 0.999$). A mini-batch size of 32 is used for training. The learning rate is initialized at 0.01 and divided by 10 at each plateau in training accuracy. The models is trained for up to 250 epochs. Our goal during the training is to minimize the weighted MSE loss function, L_w, which is calculated from the following equation

$$L_w = \sum_{n=1}^{N} \sum_{i,j,k=1}^{48} w_{ijk} \frac{(P_n(i,j,k) - G_n(i,j,k))^2}{48^3 N} \tag{2}$$

where N is the number of images, i, j, k is the voxel index, and $P_n(i,j,k)$ and $G_n(i,j,k)$ are the predicted and ground-truth ion distributions for the nth image, respectively. Further, the weights are defined as

$$w_{ijk} = \begin{cases} 1 & G_n(i,j,k) = 0 \\ 30 \cdot G_n(i,j,k) & G_n(i,j,k) \neq 0 \end{cases}$$

The above loss function gives the MSE between the predicted distributions and ground-truth distributions for all the voxels. Because the space is sparsely occupied by Mg^{2+}, the data is highly imbalanced. The weighted loss function balances the learning process by increasing the penalty of a false negative prediction for positions that are truthfully occupied by Mg^{2+}.

Training and evaluating MgNet

To perform an unbiased evaluation for MgNet, we adopt a five-fold cross-validation procedure. For each fold, we train MgNet for a total of 250 epochs with each epoch of training taking around 5 minutes. The training is conducted on 2 GTX 1080 Ti NVIDIA GPUs and one AMD Ryzen Threadripper 1950X 3.4 GHz 16-Core Processor. The loss quickly reaches a plateau and we choose the model at epoch 40 as the final model.

Clustering to predict Mg^{2+} binding sites

For an RNA with N nucleotides, we first identify the connected regions from the top-$50N$ predicted high-probability voxels around the RNA using the DBSCAN (Ester et al., 1996) clustering method. Within each high-probability region, we then use k-means clustering to generate K clusters, where K is determined from the ratio between the volume (v) of the high-density area and preset cluster size (m). We choose the representative points of the K clusters as predicted ion sites. These sites are combined and ranked based on the sum of the probabilities of all the voxels within the corresponding cluster. By changing the preset cluster size (m), we can adjust the number of clusters (K) within each high-probability region. We obtain the default clustering settings in MgNet by optimizing the performance on the validation sets through refining the preset cluster size (m=380).

Defining the evaluation metric

We use RMSD to evaluate the performance of MgNet for an individual test structure. The overall performance of the model for a large number of test structures is evaluated by the true positive rate

(TPR) and the positive predictive value (PPV). TPR and PPV are calculated from the following equations:

$$\mathrm{TPR} = \frac{\mathrm{TP}}{\mathrm{P}} = \frac{\mathrm{TP}}{\mathrm{TP} + \mathrm{FN}} \tag{3}$$

$$\mathrm{PPV} = \frac{\mathrm{TP}}{\mathrm{TP} + \mathrm{FP}} \tag{4}$$

Here, P is the number of positive (experimentally observed) cases, TP (true positive) is the number of predicted Mg^{2+} that resides within a 3 Å sphere around an experimentally observed Mg^{2+}, and FP (false positive) is the number of predicted Mg^{2+} that falls outside the 3 Å range from experimentally observed Mg^{2+} ions.

Calculating radial frequency distribution

The radial frequency distribution is generated from the following steps. First, we find all the bound Mg^{2+} ions in the training set. Second, for each Mg^{2+} in the training set, the space within 9 Å around the ion is discretized into 18 spherical shells, each having a shell thickness of 0.5 Å. For each Mg^{2+}, we locate all the RNA atoms within the 9 Å sphere and bin them in the shells. Then, according to the different types of coordinating atoms, we count the frequency of each coordinating atom type in the spherical shells for all the Mg^{2+} ions and compute the radial frequency distribution for every coordinating RNA atom type. The radial frequency in each spherical shell (or the distance bin) is normalized by the volume of the corresponding shell:

$$f_{\mathrm{t}}(i) = \frac{n_{\mathrm{t}}(i)}{v(i)} \tag{5}$$

where $n_{\mathrm{t}}(i)$ is the number of the type-t RNA atoms appearing in the ith shell for the bound Mg^{2+}, and $f_{\mathrm{t}}(i)$ is the frequency normalized by the corresponding shell volume $v(i)$.

The representative atoms are chosen by first ranking the atoms by the sum of the $n_{\mathrm{t}}(i)$ (the number of the type-t RNA atoms appearing in the ith shell) within 5 Å (the outer-sphere coordination distance) for all of atom type t and then selecting the top-ranked atom for each atom type. To differentiate the effects of the different types of atoms, we define the radial distribution of the saliency value $h_{\mathrm{t}}(i)$ as $h_{\mathrm{t}}(i) = s_{\mathrm{t}}(i)/n_{\mathrm{t}}(i)$, where $s_{\mathrm{t}}(i)$ is the sum of all the saliency values for the type-t RNA atom in the ith shell from the ion, and the denominator $n_{\mathrm{t}}(i)$ is the number of the type-t RNA atoms appearing in the ith shell. Physically, the saliency values $h_{\mathrm{t}}(i)$ indicates the relative sensitivity of ion binding site to the RNA atom types.

Supplementary figures and tables

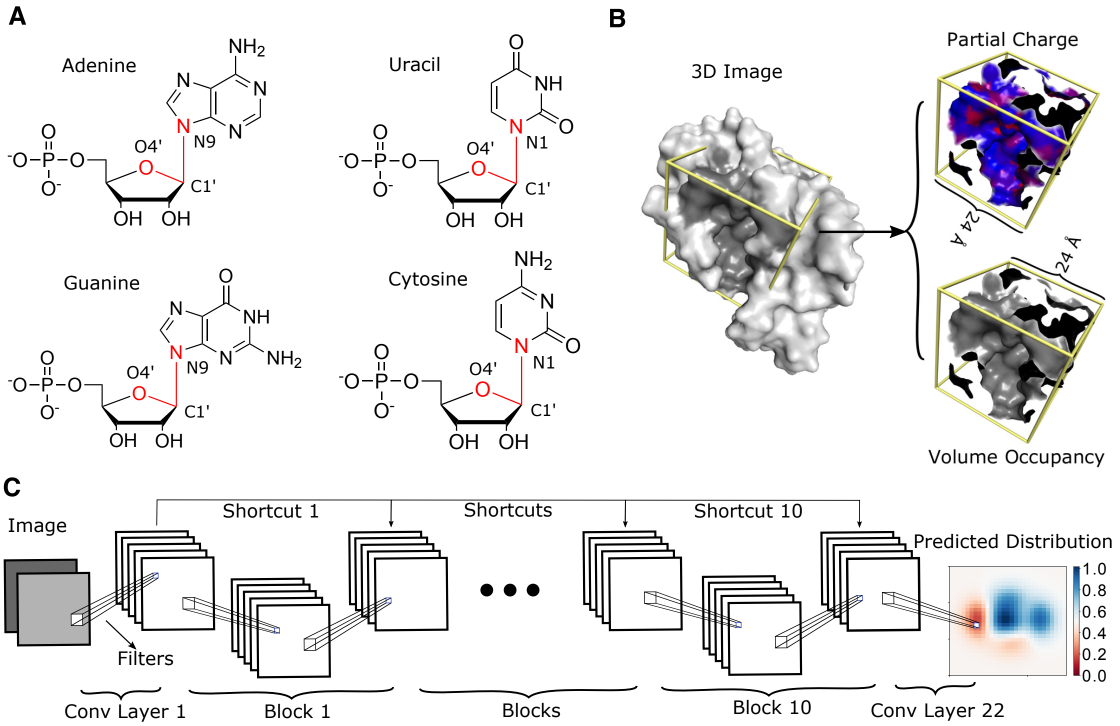

Figure S1: Overview of the MgNet. (a) To configure the 3D images for Mg^{2+} ion binding environment, we set up a local Cartesian coordinate system around each nucleotide based on three key atoms (shown in red) in the sugar ring and bases. The origin of the local coordinate system is set to the midpoint between the carbon atom (C1') and the nitrogen atom (N1 for pyrimidine or N9 for purine), where the vectors formed by C1' and an oxygen atom (O4'), and by C1' and the nitrogen atom (N1 or N9) are used to define the x-y plane of the system. (b) Each 3D image is taken from a 24 Å x 24 Å x 24 Å cubic box centered at a given nucleotide and is used to capture the information for the binding and non-binding sites. The cubic box is shown with yellow frames. The local Cartesian coordinate system (the orientation of the cubic box) is determined by the key atoms in the corresponding nucleotide. Two feature channels (partial charge and volume occupancy) are used to extract the relevant information from the image. (c) The MgNet is drawn in a 2D diagram for a better illustration, where all 3D images (3D cubic grids) are shown as 2D squares. From left to right, an image with two feature channels is fed into the MgNet, and information is then processed by different layers of filters and connected through various shortcuts. The final prediction is an ion density (probability) distribution map.

Table S1: PDBs used in five-fold cross-validation evaluation

				cv1					
1b23	1hq1	2a43	2g91	2oiu	301d	3cul	3l3c	3q51	3tzr
4l81	4qlm	4yco	5ew7	5ns4	5vjb	6b14	6cu1	1drz	1zz5
2cv1	2nok	2qus	354d	3ftm	3mei	3ssf	437d	4m30	4rge
5bjo	5ktj	5tpy	5wti	6c8d	6dta				
				cv2					
1duh	1ik5	1kxk	1nuj	2ann	2hw8	2yie	2zzn	3egz	3jxq
3loa	3ski	3v7e	4bwm	4nya	4p95	5dh6	5m0i	1feu	1j1u
1mms	1y26	2b8s	2oe5	2zzm	3cr1	3eph	3knc	3mxh	3t1y
430d	4g6r	4oji	4yb0	5lqt	5xus				
				cv3					
1jid	2nug	2qbz	3f4h	3hhn	3nd4	3oin	3u56	4frg	4m4o
4pdq	4xco	5d8h	5e54	5kpy	5ndh	5v0k	6dme	2fmt	2pjp
2val	3fs0	3ivn	3nkb	3td0	4en5	4ghl	4pcj	4pqv	4xw7
5ddp	5fj0	5mga	5u3g	5xtm					
				cv4					
1dfu	1f27	1hc8	1lnt	1mzp	1pjo	1yls	2ply	3cgs	3gvn
3la5	4oog	4znp	5btp	5lyv	5une	5y85	6dnr	1evv	1ffy
1hr2	1mji	1ntb	1y95	2g3s	364d	3d2x	3hax	3q3z	4z4f
5aox	5c9h	5t3k	5voe	6cc3					
				cv5					
1d4r	1jzv	1tra	2ao5	2q1r	2tra	3f2q	3oxd	4cs1	4jrc
4lx6	4tzx	5btm	5dar	5fj1	5u0q	6aso	6db9	1dk1	1l9a
1xjr	2fqn	2quw	2z75	3gx3	3zgz	4e8n	4k27	4rwn	4wkj
5ckk	5dhc	5nzd	5v2h	6c8o					

Table S2: TPR and PPV of the five-fold cross-validation test

	cv1	cv2	cv3	cv4	cv5	total
TPR	50.43%	49.58%	43.46%	50.19%	40.91%	46.91%
PPV	31.81%	36.53%	39.81%	38.44%	31.12%	35.54%

The table shows the TPR and PPV of the MgNet model in the five-fold cross-validation test. The column under the total is the averaged results of the five-fold cross-validation.

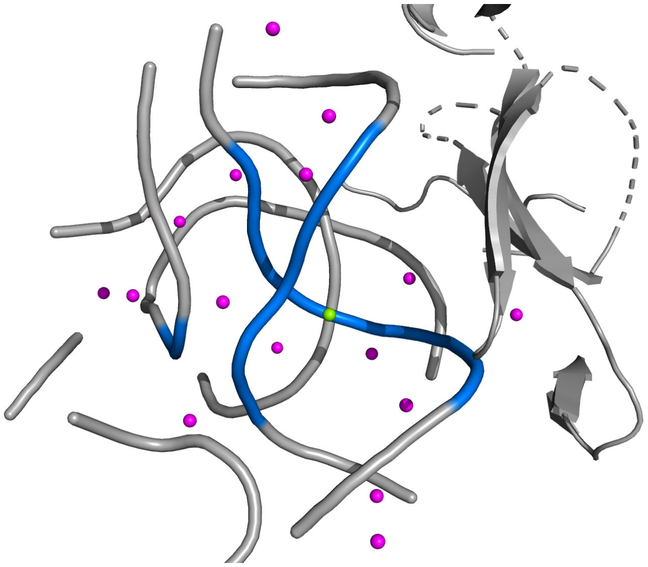

Figure S2: The neighboring environment of an experimentally observed Mg^{2+} site (green sphere) in MgRNA benchmark set. Nearby experimentally observed Mg^{2+} sites other than the benchmark sites (green sphere) are shown as magenta spheres. Only 3D images associated with nearby nucleotides (shown in blue) within 10 Å of the MgRNA benchmark Mg^{2+} sites are used by MgNet to predict ion distributions. Although the remaining residues (shown in grey, including RNA and protein segments) do not have associated images, they are still covered by various generated images centered at nearby nucleotides (shown in blue).

Table S3: Success rate of MgNet model on the high-quality dataset

	cv1	cv2	cv3	cv4	cv5	total
TPR	51.90% (205/395)	47.85% (189/395)	49.62% (196/395)	45.57% (180/395)	49.49% (195/394)	48.89% (965/1974)

The TPR (i.e., success rate) of five-fold MgNet models on the high-quality dataset. Columns cv1 to cv5 show the predictions made by the corresponding trained MgNet models. Only predicted sites with RMSD less than 3 Å are considered as true positive ones. The column under the total is the averaged results of the five-fold cross-validation. The numbers in parenthesis represent the number of the correct predictions and the number of the experimental sites, respectively.

Table S4: Comparison between the performance of MetalionRNA and of MgNet for the 58 nt fragment of 23S rRNA structure (PDB code: 1HC8)

Mg^{2+} (res no.)	MetalionRNA(29) Å(rank)	MgNet(9) Å(rank)	*MgNet(12) Å(rank)
1159	0.8 (1)	0.8 (8)	0.8 (7)
1160	1.9 (6)	-	0.6 (12)
1161	2.9 (29)	2.8 (7)	2.3 (8)
1163	0.6 (3)	1.8 (5)	1.3 (4)
1164	1.4 (2)	2.3 (3)	1.4 (5)
1167	3.8 (10)	0.5 (1)	0.5 (3)
1172	3.2 (13)	1.6 (9)	1.8 (9)

RMSD values and ranks of the predictions of MetalionRNA and MgNet for Mg^{2+} ions in the 58 nt fragment of 23S rRNA structure (PDB code: 1HC8). The leftmost column lists the Mg^{2+} identifiers (residue number) as labeled in the PDB file. From the second column to the rightmost column, we summarize the predictions made by MetalionRNA, MgNet with default cluster setting, and MgNet with an adjusted cluster setting, respectively (see Supplementary Information). The 7 experimentally determined ion sites are successfully predicted by MetalionRNA, MgNet, and *MgNet within the top-29, 9, and 12 ranked hits, respectively. For each entry, the number in parenthesis indicates the rank of the corresponding prediction. Lower ranked sites correspond to predicted sites with lower confidence. A dashed line means there is no predicted ion for the corresponding experimental binding site. Among the top-9 predictions from MgNet, six out of the seven experimentally observed Mg^{2+} ions are predicted with an accuracy of 0.5-2.8 Å. The remaining experimental ion is found in between two experimentally determined ions (residue numbers 1161 and 1160) with a distance of 2.8 Å and 3.6 Å to the ions of residue numbers 1161 and 1160, respectively. The result suggests that these two Mg^{2+} sites may share a mutual binding area. As shown in the *MgNet column, the top-12 predicted Mg^{2+} ion coordinates give all the seven experimentally determined ions with an accuracy of 0.5-2.3 Å.

Table S5: Comparison between the molecular dynamics (MD) simulation-based method and MgNet for seven test structures

PDB	Ion	Mg^{2+}_{CI}	Mg^{2+}_{PS}	MgNet
**1D4R**	MG-90	1.0 ± 0.5	1.1 ± 0.5	2.2 ± 0.6
	MG-91	5.0 ± 0.7	4.4 ± 0.7	3.7 ± 0.8
**2MTK**	MG-48	7.4 ± 3.2	5.8 ± 1.9	4.8 ± 0.5
	MG-49	3.9 ± 0.9	2.9 ± 1.6	3.6 ± 0.5
	MG-50	6.7 ± 3.0	5.7 ± 2.3	1.6 ± 0.2
	MG-51	3.2 ± 0.8	3.5 ± 0.4	7.1 ± 8.1
	MG-52	3.6 ± 0.5	3.8 ± 2.4	7.7 ± 0.4
	MG-53	2.1 ± 0.4	2.3 ± 1.1	2.1 ± 0.7
**2QEK**	MG-49	2.5 ± 0.2	2.5 ± 1.3	6.3 ± 4.4
**4FRG**	MG-179	2.4 ± 0.7	4.4 ± 0.8	1.2 ± 0.6
	MG-180	2.4 ± 0.8	5.3 ± 0.4	1.5 ± 0.4
	MG-181	2.8 ± 0.5	1.4 ± 0.5	18.8 ± 0.2
	MG-182	7.6 ± 0.5	7.0 ± 0.6	2.0 ± 0.1
	MG-183	3.7 ± 1.5	4.7 ± 2.9	1.6 ± 0.8
	MG-184	1.1 ± 0.3	2.0 ± 1.5	2.1 ± 0.9
	MG-185	3.7 ± 1.3	5.9 ± 1.4	4.8 ± 5.5
**4JF2**	MG-94	2.2 ± 1.1	2.7 ± 1.0	0.7 ± 0.2
	MG-95	3.2 ± 0.6	4.6 ± 0.7	4.0 ± 6.7
	MG-96	2.5 ± 0.8	2.9 ± 0.9	0.5 ± 0.1
	MG-97	18.3 ± 2.7	20.6 ± 0.8	0.6 ± 0.2
**4KQY**	MG-121	1.8 ± 0.4	3.4 ± 0.9	1.3 ± 0.4
	MG-122	1.8 ± 0.5	4.2 ± 2.0	6.4 ± 1.4
**4P5J**	MG-85	1.1 ± 0.7	1.8 ± 0.2	1.5 ± 0.4
	MG-86	2.5 ± 0.5	3.5 ± 2.1	1.3 ± 0.1

RMSD values and the standard deviations between the predicted ion sites and the corresponding experimental ion sites, measured in angstrom. The PDB code and the corresponding experimental Mg^{2+} ions are listed in the first two columns. Column Mg^{2+}_{CI} and Mg^{2+}_{PS} show the average RMSD values and the standard deviations of MD simulation-based method. The top-50 predicted sites from the MD simulation-based method are used. Column MgNet shows the averaged RMSD values over the predictions of the five trained MgNet models. We note that MD simulation-based method do not provide the rank order for the predicted ions, thus we only list the average RMSD and the standard deviation for each Mg^{2+}. Details of the predictions of MgNet model can also be found in Supplementary Table S6 and Supplementary Table S7.

Table S6: RMSD table of the MD simulation-based method and MgNet on seven test structures

PDB	Ion	Mg^{2+}_{CI}	Mg^{2+}_{PS}	cv1	cv2	cv3	cv4	cv5
**1D4R**	MG-90	1.0 ± 0.5	1.1 ± 0.5	2.9 (1)	2.4 (1)	1.6 (1)	1.6 (2)	2.3 (1)
	MG-91	5.0 ± 0.7	4.4 ± 0.7	2.8 (4)	5.0 (2)	3.4 (2)	3.6 (1)	3.5 (2)
**2MTK**	MG-48	7.4 ± 3.2	5.8 ± 1.9	4.9 (1)	5.2 (7)	4.7 (7)	3.9 (2)	5.2 (5)
	MG-49	3.9 ± 0.9	2.9 ± 1.6	3.1 (6)	4.0 (5)	4.1 (8)	3.7 (7)	3.0 (8)
	MG-50	6.7 ± 3.0	5.7 ± 2.3	1.3 (2)	1.5 (1)	1.8 (4)	1.8 (3)	1.5 (2)
	MG-51	3.2 ± 0.8	3.5 ± 0.4	19.5 (4)	1.5 (8)	11.5 (3)	1.2 (1)	2.0 (9)
	MG-52	3.6 ± 0.5	3.8 ± 2.4	7.6 (3)	8.0 (3)	7.6 (1)	8.2 (5)	7.2 (6)
	MG-53	2.1 ± 0.4	2.3 ± 1.1	2.0 (7)	1.6 (2)	3.2 (9)	1.5 (4)	2.0 (1)
**2QEK**	MG-49	2.5 ± 0.2	2.5 ± 1.3	1.5 (4)	1.6 (6)	9.8 (1)	8.9 (4)	9.9 (1)
**4FRG**	MG-179	2.4 ± 0.7	4.4 ± 0.8	1.5 (2)	0.9 (2)	1.8 (1)	1.5 (1)	0.4 (6)
	MG-180	2.4 ± 0.8	5.3 ± 0.4	0.9 (4)	1.7 (8)	1.4 (8)	1.6 (5)	1.9 (5)
	MG-181	2.8 ± 0.5	1.4 ± 0.5	18.6 (5)	18.9 (11)	-	-	18.8 (10)
	MG-182	7.6 ± 0.5	7.0 ± 0.6	1.8 (10)	2.0 (7)	1.9 (11)	2.1 (4)	-
	MG-183	3.7 ± 1.5	4.7 ± 2.9	2.2 (1)	2.5 (1)	0.7 (5)	2.0 (2)	0.8 (2)
	MG-184	1.1 ± 0.3	2.0 ± 1.5	1.2 (3)	3.4 (3)	2.1 (10)	1.3 (6)	2.6 (1)
	MG-185	3.7 ± 1.3	5.9 ± 1.4	1.1 (9)	0.8 (10)	7.5 (6)	13.3 (7)	1.4 (3)
**4JF2**	MG-94	2.2 ± 1.1	2.7 ± 1.0	0.4 (3)	0.7 (1)	0.8 (4)	0.9 (6)	0.5 (1)
	MG-95	3.2 ± 0.6	4.6 ± 0.7	16.0 (5)	1.1 (8)	1.1 (2)	1.0 (4)	1.0 (4)
	MG-96	2.5 ± 0.8	2.9 ± 0.9	0.4 (7)	0.6 (4)	0.6 (3)	0.5 (7)	0.5 (5)
	MG-97	18.3 ± 2.7	20.6 ± 0.8	0.7 (2)	0.7 (2)	0.9 (1)	0.6 (1)	0.3 (3)
**4KQY**	MG-121	1.8 ± 0.4	3.4 ± 0.9	1.9 (1)	1.3 (3)	1.2 (5)	0.7 (1)	1.4 (1)
	MG-122	1.8 ± 0.5	4.2 ± 2.0	7.1 (2)	5.4 (8)	5.7 (8)	5.3 (6)	8.5 (7)
**4P5J**	MG-85	1.1 ± 0.7	1.8 ± 0.2	1.6 (5)	1.1 (1)	1.4 (6)	1.2 (2)	2.0 (1)
	MG-86	2.5 ± 0.5	3.5 ± 2.1	1.3 (6)	1.3 (2)	1.4 (2)	1.3 (1)	1.2 (3)

Column ${Mg^{2+}}_{CI}$ and ${Mg^{2+}}_{PS}$ are the average RMSD values and standard deviations of MD method between experimental and predicted binding sites during the production phase. Top 50 predicted sites were used in MD method. Columns cv1 to cv5 are the predictions made by MgNet with default clustering settings, shown in RMSD values with ranks in parentheses. In MgNet model, only predicted sites with RMSD less than 20 Å are listed in the table, experimental ions with no predicted sites within 20 Å are labeled with dash.

Table S7: TPR and PPV of MD simulation-based method and MgNet on seven test structures

	Mg^{2+}_{CI}	Mg^{2+}_{PS}	cv1	cv2	cv3	cv4	cv5
TPR	-	-	70.83%	70.83%	58.33%	66.67%	70.83%
PPV	-	-	31.48%	26.98%	25.93%	29.63%	32.08%
TPR*	54.17%	37.50%	87.50%	87.50%	87.50%	91.67%	91.67%
PPV*	3.71%	2.57%	6.00%	6.00%	6.00%	6.29%	6.29%

The TPR and PPV for both MD simulations and MgNet. Column Mg^{2+}_{CI} and Mg^{2+}_{PS} are the results of MD simulations with the different ion conditions. Columns cv1 to cv5 are the predictions made by the corresponding trained MgNet models. Only predicted sites with RMSD less than 3 Å are considered as true positive ones. For MD simulations, predictions were made by using the top 50 predicted sites. Since the default clustering settings of MgNet tend to give fewer predictions than the MD simulations (i.e., 50 sites), we provide MgNet results with two different settings. One uses default clustering settings and the other one uses the same number of predicted binding sites for each structure as the MD simulations (i.e., 350 predicted sites for seven structures). The results of these two settings are shown as TPR and PPV without and with asterisk, respectively).

Table S8: Comparison between the Brownian dynamics (BD) simulation-based method and MgNet for three test structures

PDB	Ion	BD	cv1	cv2	cv3	cv4	cv5
**354D**	A-203	1.8	0.9 (2)	1.1 (2)	1.6 (6)	1.2 (7)	1.0 (3)
	B-200	0.7	0.4 (1)	0.5 (3)	0.5 (2)	0.9 (1)	1.0 (2)
	B-201	1.3	1.2 (4)	1.6 (5)	1.3 (4)	1.0 (5)	0.9 (1)
	B-202	1.4	1.1 (5)	1.1 (1)	1.7 (3)	0.8 (4)	1.2 (6)
	B-204	2.7	1.4 (7)	1.6 (6)	5.8 (1)	1.6 (6)	6.4 (4)
**3TRA**	A-76	$\sim$5.0	-	-	-	4.3 (6)	-
**4TRA**	A-77	2.6	2.0 (4)	2.5 (6)	2.1 (7)	2.4 (4)	2.2 (4)
	A-78	2.1	1.0 (5)	0.7 (2)	0.9 (4)	0.5 (5)	1.4 (1)
	A-79	2.2	2.1 (7)	1.2 (3)	1.0 (5)	1.8 (6)	1.9 (5)
	A-80	0.3	6.2 (6)	-	-	6.1 (7)	6.0 (6)

RMSD values between the predicted ion sites and the experimental ion sites, measured in angstrom. The PDB codes and the corresponding experimental Mg^{2+} ions are listed in the first two columns. From the simulation of many positively charged spheres under the influence of both random Brownian motion and the electrostatic field of the RNA, the binding sites are identified as the regions where a significant number of the test charges are trapped. The results of the trained MgNet models are listed from columns cv1 to cv5 with the ranks shown in parentheses. Experimentally determined ion sites that theoretical models fail to predict within 10 Å are labeled with a dash.

Table S9: Comparison between the performance of MgNet model for the original RNAs and for RNAs with the coordinating atoms removed

Mg^{2+}	RNA	RNA^R
2YIE-Z1116	1/1	0/1
3HAX-E200	1/1	1/1
3Q3Z-V85	1/1	0/1
2Z75-B301	1/1	0/1
3DD2-B1000	5/5	2/5
1VQ8-08004	5/5	0/5
4TP8-A1601	3/5	0/5
2QBA-B3321	5/5	0/5

The number of successful MgNet predictions for each Mg^{2+} binding case. Predictions are made by five previously trained models obtained through five-fold cross-validation. However, for a binding case included in the cross-validation dataset (top-four cases), only the model trained without the case is used to make predictions so the test set is not included in the training set. The purpose of this test is to show the importance of the coordinating atoms – the removal or change of the coordinating atoms would result in incorrect binding sites. The first column shows the PDB code and the Mg^{2+} identifier for the position of the bound ion. The column labeled with RNA and RNA^R shows the result for the original RNA and the RNA with coordinating atoms removed, respectively. The results are shown in the n/N format, where n and N represent the numbers of the MgNet model with successful predictions and of all the trained MgNet models, respectively. A prediction is successful if the RMSD between the predicted ion site and the experimentally observed site is within 3 Å.

Table S10: Feature channels used for the 3D descriptor

Feature	Rule
volume occupancy	all the RNA atom types (not including Mg^{2+})
partial charge	partial charge values for all the RNA atom types (not including Mg^{2+})

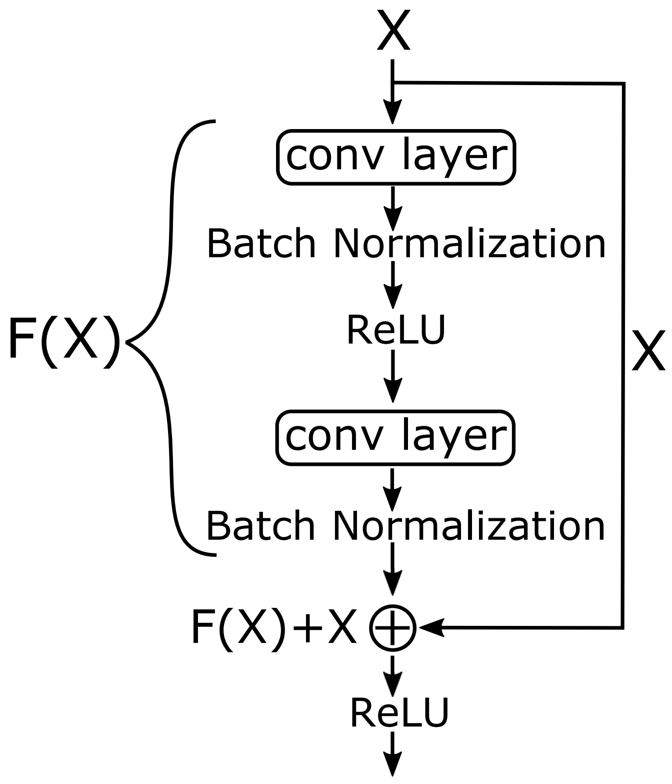

Figure S3: Block structure with a residual shortcut. Figure shows the entire block structure, where X is the input of this block (i.e., X is the output from the previous layer) and ReLU is the Rectified Linear Unit. Within this block, input X passes through two convolutional layers. The whole transformation in this block can be viewed as a function F, which maps input X to output F(X), and an identity-mapping shortcut on the right-hand side adds X directly to the processed output F(X).

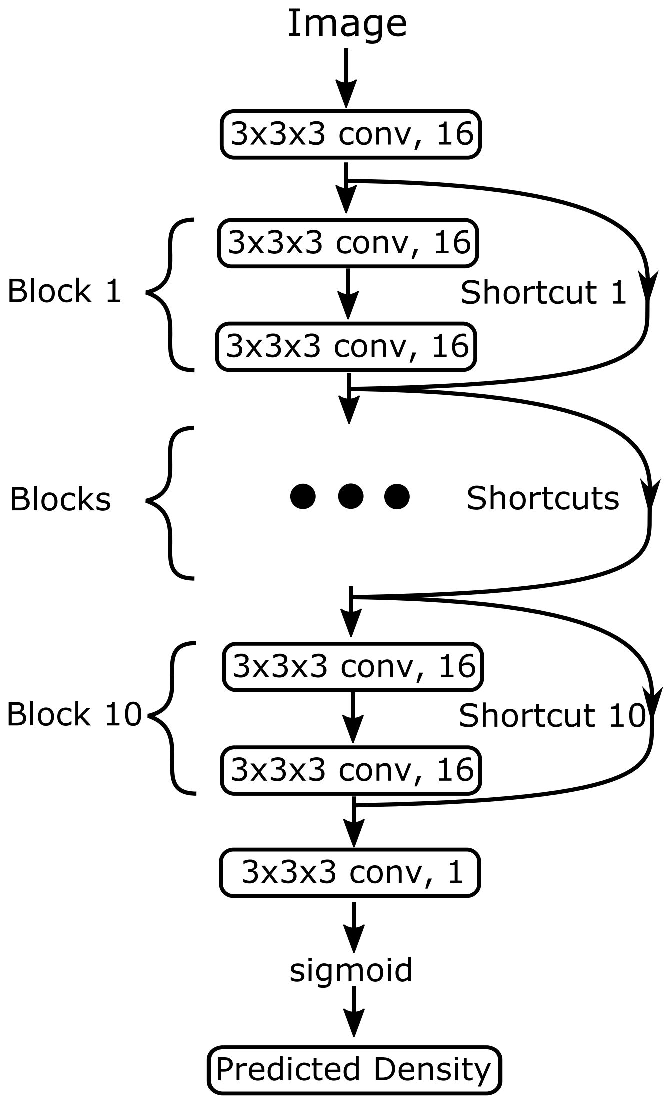

Figure S4: MgNet model. Ten blocks are stacked sequentially to make a 22-layer CNN. All convolutional layers have the same number of filters except for the last layer, which only has one filter. A sigmoidal activation function is applied to confine the predicted ion density within the $0 \sim 1$ range.

Table S11: Details of MgNet Architecture

Block	Layer	Output Size	Filter size	Filter number	Padding
first conv	conv1	$48 \times 48 \times 48$	$3 \times 3 \times 3$	16	1
block1	conv2	$48 \times 48 \times 48$	$3 \times 3 \times 3$	16	1
	conv3	$48 \times 48 \times 48$	$3 \times 3 \times 3$	16	1
···	···	···	···	···	···
block10	conv20	$48 \times 48 \times 48$	$3 \times 3 \times 3$	16	1
	conv21	$48 \times 48 \times 48$	$3 \times 3 \times 3$	16	1
last conv	conv22	$48 \times 48 \times 48$	$3 \times 3 \times 3$	1	0

Architectures of MgNet. Each building block is shown with two convolutional layers together without line separation. No downsampling is performed in this network, so the stride has size equal to 1 for all layers.

Dataset S1: SI_sheet.xlsx (a separate file)
Contains information about the datasets, cross-validation, comparisons with other methods, and the identified binding motifs.

References

Doerr, S., Harvey, M. J., Noé, F., and De Fabritiis, G. (2016). Htmd: High-throughput molecular dynamics for molecular discovery. *Journal of Chemical Theory and Computation*, 12(4):1845–1852. PMID: 26949976.

Ester, M., Kriegel, H.-P., Sander, J., Xu, X., et al. (1996). A density-based algorithm for discovering clusters in large spatial databases with noise.

He, K., Zhang, X., Ren, S., and Sun, J. (2015). Spatial pyramid pooling in deep convolutional networks for visual recognition. *IEEE Transactions on Pattern Analysis and Machine Intelligence*, 37(9):1904–1916.

He, K., Zhang, X., Ren, S., and Sun, J. (2016). Deep residual learning for image recognition. In *Proceedings of the IEEE Conference on Computer Vision and Pattern Recognition (CVPR)*.

Ioffe, S. and Szegedy, C. (2015). Batch normalization: Accelerating deep network training by reducing internal covariate shift. In Bach, F. and Blei, D., editors, *Proceedings of the 32nd International Conference on Machine Learning*, volume 37 of *Proceedings of Machine Learning Research*, pages 448–456, Lille, France. PMLR.

Kingma, D. P. and Ba, J. (2017). Adam: A method for stochastic optimization.

Leontis, N. B. and Zirbel, C. L. (2012). Nonredundant 3d structure datasets for rna knowledge extraction and benchmarking. In *RNA 3D structure analysis and prediction*, pages 281–298. Springer.

Nair, V. and Hinton, G. E. (2010). Rectified linear units improve restricted boltzmann machines. In *ICML*, pages 807–814.

Paszke, A., Gross, S., Massa, F., Lerer, A., Bradbury, J., Chanan, G., Killeen, T., Lin, Z., Gimelshein, N., Antiga, L., Desmaison, A., Kopf, A., Yang, E., DeVito, Z., Raison, M., Tejani, A., Chilamkurthy, S., Steiner, B., Fang, L., Bai, J., and Chintala, S. (2019). Pytorch: An imperative style, high-performance deep learning library. In Wallach, H., Larochelle, H., Beygelzimer, A., d'Alché-Buc, F., Fox, E., and Garnett, R., editors, *Advances in Neural Information Processing Systems*, volume 32. Curran Associates, Inc.

Zheng, H., Shabalin, I. G., Handing, K. B., Bujnicki, J. M., and Minor, W. (2015). Magnesium-binding architectures in RNA crystal structures: validation, binding preferences, classification and motif detection. *Nucleic Acids Research*, 43(7):3789–3801.
